# Iron and zinc-modified nano-biochar alleviate salinity stress in paddy soil by modulating nutrient availability and microbial communities

**DOI:** 10.3389/fmicb.2025.1729271

**Published:** 2026-01-16

**Authors:** Asad Shah, Haider Sultan, Mohammad Nauman Khan, Waqar Ali, Hafiz Muhammad Mazhar Abbas, Muhammad Numan Khan, Tao Ye, Lixiao Nie

**Affiliations:** 1 Sanya National Center of Technology Innovation for Saline-Alkali Tolerant Rice, School of Breeding and Multiplication (Sanya Institute of Breeding and Multiplication), Hainan University, Sanya, China; 2 Center for Eco-Environment Restoration Engineering of Hainan Province, School of Ecology, Hainan University, Haikou, China; 3 Yunnan Key Laboratory of Forest Ecosystem Stability and Global Change, Xishuangbanna Tropical Botanical Garden, Chinese Academy of Sciences, Mengla, Yunnan, China

**Keywords:** bacterial community, fungal abundance, nano-biochar, salinity, soil enzymes, soil physiochemical properties

## Abstract

**Introduction:**

Soil salinization is a major constraint to agricultural productivity on Hainan Island, China, as it reduces soil fertility and disrupts microbial community structure. Nano-modified biochar has emerged as a promising strategy to improve soil quality and microbial resilience under saline conditions. This study evaluated the effects of iron- and zinc-modified nano-biochar on soil physicochemical properties and microbial communities in saline and non-saline paddy soils.

**Methods:**

A pot experiment was conducted using four treatments: control (CK), pristine biochar (BC), iron-modified biochar (FeBC), and zinc-modified biochar (ZnBC). Soil physicochemical properties, enzyme activities, microbial biomass, and bacterial and fungal community composition were analyzed using standard chemical assays and high-throughput sequencing techniques.

**Results:**

Application of nano-modified biochar significantly increased soil organic matter, soil organic carbon, and the availability of macronutrients (N, P, and K). FeBC and ZnBC enhanced microbial biomass carbon and nitrogen as well as urease and β-glycosidase activities in saline soil. Nano-biochar treatments altered microbial community composition, increasing the abundance of salt-tolerant bacterial phyla such as *Proteobacteria*, *Chloroflexi*, and *Bacteroidota* under saline conditions, while modifying fungal community structure, including increased relative abundance of Chytridiomycota under FeBC treatment.

## Introduction

Salinization is a major abiotic stress limiting crop production worldwide. Saline soil accelerates soil degradation by disrupting the soil structure, reducing nutrient availability, hindering water absorption, and ultimately decreasing agricultural productivity and crop yields (Cuevas et al., 2019). Irrigation mismanagement, soil deterioration, and climate change are contributing to the growing amount of salinity affected soils (Suleymanov et al., 2023), which in turn complicates agricultural and ecosystem management (Faizan et al., 2023). Approximately 833 million hectares which is 8.7% of the global land area are saline soil, which mostly occurring in arid and semi-arid environments. Saline soil covers an area of approximately 1.1 × 10^9^ hm^2^ worldwide and is continuously expanding due to natural processes and human activity (Guo et al., 2018; Ma and Tashpolat, 2023). In China, saline soils cover approximately 3.69 × 107 hm^2^, accounting for nearly 4.88% of the country’s total land area, and paddy soil is a key component of cropland and a vital resource for food security (Xin et al., 2020; Dewi et al., 2022). However, paddy soil has been severely degraded due to prolonged and improper fertilizer application, further exacerbating this degradation by inhibiting water retention, reducing soil fertility, limiting nutrient availability, and ultimately lowering crop yields (Ye et al., 2022; Srivastava et al., 2024). Thus, adopting suitable management practices to reduce soil salinization can protect soil resources and ensure long-term sustainable agricultural productivity and environmental quality.

Biochar is a carbon rich material, which is manufactured through the process of pyrolysis, and which involves heating biomass in oxygen restricted conditions (Wang et al., 2023). Biochar contains abundant nutrients, and can supply essential mineral elements to plants and alleviating salinity stress. When biochar is applied to saline soil, it can promote plant growth by improving physical structure, preserving moisture and nutrients, and reducing salinity level. Nevertheless, some studies have showed that the alkalinity nature of biochar limits its benefits in salt affected soils. Therefore, modifying biochar is a novel strategy to improve the performance of biochar in specific environments. Nanobiochar, characterized by its high surface area and strong zeta potential, offers significant potential for remediating saline soils and enhancing agricultural sustainability (He et al., 2020; Singh et al., 2024; Wang et al., 2024). Its application mitigates salinity stress, improves soil structure, and increase crop yields by enhancing water retention, pore size, and tensile strength, thereby increasing soil fertility (Guo et al., 2018; Tarolli et al., 2024; Zhang et al., 2024). For instance, the application of approximately 8% nano biochar has been shown to increase the soil organic carbon content by 41%, highlighting its role in stabilizing soil carbon and promoting long-term soil health (Hussein et al., 2022). Nanobiochar enhances soil structure, thereby creating optimal conditions for soil microbial communities, which play a critical role in maintaining soil fertility and supporting crop productivity (Hardy et al., 2019). High salt concentrations typically disrupt microbial activity and community composition; however, nanobiochar counteracts these effects by optimizing soil pH, adhesion, and nutrient availability, including dissolved organic matter, phosphorus, and nitrogen (Zhang et al., 2024; Ighalo et al., 2025). Its porous structure supplies essential nutrients and degradable organic metabolites, supporting microbial growth and processes such as nitrogen and phosphorus cycling and organic matter decomposition (Sani et al., 2023; Ighalo et al., 2025). Nanobiochar improves soil physicochemical properties and microbial dynamics, fostering a balanced ecosystem that promotes sustainable agricultural productivity.

Soil biochemical and microbial properties serve as sensitive bioindicators of salinity-induced stress in agroecosystems (Azadi and Raiesi, 2021). Nanobiochar influences microbial growth, alters soil physicochemical properties; and significantly affects microbial diversity, structure, and abundance. Salt stress typically reduces bacterial diversity and enzyme activity, but biochar enhances microbial activity and nutrient cycling by increasing aeration and organic carbon availability (Ali et al., 2021). Nanobiochar elicits species specific responses, with notable increases in Actinobacteria and Bacteroidetes biomass following the application of wheat straw nanobiochar application (Kaoping et al., 2019; Zhao et al., 2020). The application of nanobiochar elicited varied responses from different bacterial and fungal species, highlighting species-specific interactions. When Fe-Mn-modified biochar was applied, the bacterial phyla *Firmicutes* and *Proteobacteria* also showed dominant responses (Shen et al., 2013). When Fe-Mn-Ce-modified biochar (formed by combining the 3 elements of iron, manganese, and cerium with biochar) was applied, the *Gemmatimonadaceae* family within the phylum *Gemmatimonadetes* and the *Oxalobacteraceae* family within the phylum *Proteobacteria* showed dominant responses as the activity levels of these microbes increased within the soil (Zhang et al., 2020). At the genus level, the abundances of *Gemmatimonas* and *Sphingomonas* predominantly increased, leading to enhanced nitrogen transformation when nano-zero-valent iron was applied (Liu et al., 2021). In addition to Fe-Mn-La-modified biochar (formed by combining the 3 elements iron, manganese, and lanthanum with biochar), the bacterial phylum *Proteobacteria*, specifically the γ*-Proteobacteria* and α*-Proteobacteria* subgroups, and the phylum *Acidobacteria* showed a dominant effect as the activity of these microbes increased within the soil.

The effect of biochar on fungal diversity and community structure are well-documented, yet the dominant fungal phyla, Ascomycota and Basidiomycota, responded minimally to biochar application (Dai et al., 2021). At the bacterial phylum level, the activity of Proteobacteria decreased with the addition of biochar (Brar et al., 2024), and the abundance of Bacteroidetes decreased with the addition of Fe-Mn-modified biochar; however, the underlying mechanisms remain unclear. Therefore, it is imperative to study the effect of biochar and nanomodified biocharon the microbial community to (1) investigate the effects of Fe and Zn nanobiochar on bacterial and fungal communities at the phylum and genus levels under saline soil conditions and. (2) examine the impact of biochar and nanomodified biochar on soil nutrients and their interactions with microbial communities in both SS and NS soils.

## Material and methods

### Biochar modification and preparation

Biochar (BC) was purchased from Henan Lize Environmental Technology Co., Ltd. The raw BC was purified by washing with ultrapure water (resistivity: 18.2 MΩ cm) until a neutral pH was achieved, followed by oven-drying at 80°C. The dried, neutralized BC was modified following the methodology of using Fe2O3 and ZnO at a concentration of 20 mg L^–1^. Specifically, 250 g of BC was sieved to a particle size of < 0.25 μm and suspended in ultrapure water containing the nanomaterials. The weight ratio of the nanomaterial solution to BC was set at 1:40. The suspension was ultrasonically agitated for 2 h, then oven-dried at 80°C. Subsequently, the BC-nanomaterial mixture was pyrolyzed at 600°C for 30 min under a nitrogen (N2) atmosphere to produce nano-biochar. The obtained samples were thoroughly rinsed with deionized water to remove residual impurities and dried at 80°C. The overall modification process of raw biochar with nanoparticles was adapted (Ghassemi-Golezani and Rahimzadeh, 2022). Each 5 kg of pot received 50 g of nanomodified biochar and biochar.

### Research site and experimental design

A pot experiment was conducted in a randomized block design with three replicates during the early sowing season of 2023 under greenhouse conditions at Hainan University, China. Topsoil (0–20 cm) was collected from farmland of Lingao County near Haikou, China (19° 34’-20° 02’ N, 109° 3’ -109° 53’E). Four treatments; control (CK), biochar (BC), iron-modified biochar (FeBC), and zinc-modified biochar (ZnBC) Nano-modified biochar, amended with Fe2O3 and ZnO, was incorporated into the soil before sowing rice seedlings. The plants were irrigated with freshwater for 25 days, from germination to the tillering stage. A basal dose of 2 g of compound fertilizer was applied to the soil. After the tillering Stage, the plant was irrigated with saline water (0.6% NaCl) and the solution was applied gradually to the pots to avoid osmotic shock and to maintain uniform salinity throughout the experiment. The salt levels in the pots were measured using a salinometer (WS-200 PLUS). Crop management practices were consistently applied under local agricultural standards. In each pot, six rice seedlings were initially transplanted to ensure uniform establishment and survival. The total duration of the experiments was 80 days and Crop was harvested before reproductive maturity. After proper establishment (approximately 1 week after transplanting), the number of seedlings was thinned to four per pot to maintain uniform plant density and minimize competition for nutrients, water, and light. In this study, the standard agronomic practices for rice cultivation under saline conditions were followed. A basal dose of fertilizers was applied at the rate of 120 kg N, 60 kg P_2_O_5_, and 40 kg K_2_O per hectare, with nitrogen supplied in three equal splits. Irrigation was maintained with non-saline water to keep a 2–3 cm water depth throughout the growth period, except during the salinity stress period when saline solution was applied to maintain the desired electrical conductivity levels. Regarding plant protection, no severe pest or disease outbreak was recorded during the experiment; however, a preventive fungicide spray of Carbendazim (0.1%) was applied at the tillering stage to minimize the risk of fungal infection. These details have been incorporated into the Materials and Methods section of the revised manuscript After harvesting the rice crop from the pots, soil sample were collected from this soil. The soil, which was initially Coastal sandy soil, is a typical ultisol with low fertility. Its pH is 6.07, and its OM content is 3.02 g/kg. In the original soil, TN, TP, TK, AP, AK, Nitrate N and Ammonium N in the original soil were 0.25 g kg, 0.31 g/kg, 0.88 g/kg, 7.2 mg/kg, 22.6 mg/kg, 1.25 mg/kg and 2.3 mg/kg, respectively.

### Soil sample analysis

Soil samples were collected from each pot and stored in plastic bags. A subsample of fresh soil was preserved at -80°C for enzyme activity analysis and DNA extraction, while the remaining soil was air-dried and sieved for chemical analysis. Soil chemical properties were assessed using standard methods. Soil pH was measured in a 1:2.5 soil-to-deionized water ratio using a pH meter (Mettler Toledo 320-S, Switzerland) following Inyang et al. (2012) (Inyang et al., 2012). SOM was measured by applying the K_2_Cr_2_O_7_ volumetric method. Soil organic carbon (SOC) was determined by using the wet digestion method. Total nitrogen in biochar samples was analyzed with a CN analyzer (Vario Max, Elementar, Germany), while total phosphorus (TP) and total potassium (TK) were quantified via wet digestion (H_2_SO_4_-HClO_4_). Available phosphorus (AP) was extracted using 0.5 M sodium bicarbonate (NaHCO_3_) and measured via Mo-Sb spectrophotometry, whereas available potassium (AK) was determined using a 0.5 M ammonium acetate (NH4OAc) extraction method. Ammonium (NH_4_^+^) and nitrate (NO_3_^–^) concentrations were analyzed from fresh soil samples extracted with 2 M KCl (1:10) and quantified using a San + + Continuous Flow Analyzer (Skalar, Netherlands). For urease enzymes, we used the method described by Yang et al. (2016). The activities of catalase (CAT) and β-glycosidase were measured according to the methods described by Tabatabai (1994). The catalase activity was determined based on the recovery of hydrogen peroxide (H_2_O_2_). Briefly, 2 g of air-dried soil samples were treated with 0.3% H_2_O_2_, with the resulting filtrate being subsequently titrated with 0.1 mol/L KMnO_4_ after 20 min of reaction (Guan et al., 1986). For the determination of microbial biomass phosphorus (MBP), nitrogen (MBN), and carbon (MBC), we used the fumigation method (Brookes et al., 1982).

### DNA extraction and PCR amplification

The soil samples were processed to extract total microbial genomic DNA via the E.Z.N.A.^®^ Soil DNA Kit (Omega Bio-Tek, Norcross, GA, United States). DNA quality and concentration were determined by 1.0% agarose gel electrophoresis and measurement with a NanoDrop 2000 spectrophotometer (Thermo Scientific), followed by storage at -80°C for subsequent analysis. The hypervariable region of the bacterial 16S rRNA gene was amplified with the primer pairs 338F (5’-ACTCCTACGGGAGGCAGCAG-3’) and 806R (5’-GGACTACHVGGGTWTCTAAT-3’) (Liu et al., 2016) via a T100 Thermal Cycler PCR system (Bio-Rad, United States). The PCR mixture was composed of 4 μL of 5 × fast buffer, 2 μL of 2.5 mM dNTPs, 0.8 μL of each primer (5 μM), 0.4 μL of Fast Pfu polymerase, 10 ng of template DNA, and ddH2O, adjusted to a total volume of 20 μL. The amplification protocol started with initial denaturation at 95°C for 3 min, followed by 27 cycles of denaturation at 95°C for 30 s, annealing at 55°C for 30 s, and extension at 72°C for 45 s. A final extension was performed at 72°C for 10 min, and the reaction mixture was then stored at 4°C. The PCR mixture was prepared by combining 4 μL of 5 × Fast Pfu buffer, 2 μL of 2.5 mM dNTPs, 0.8 μL of each 5 μM primer, 0.4 μL of Fast Pfu polymerase, 10 ng of template DNA, and ddH2O to achieve a final volume of 20 μL. The disinfected amplicons were combined at equimolar concentrations and then sequenced via paired-end sequencing on the Illumina NextSeq 2000 platform (San Diego, United States) according to the standard protocols provided by Majorbio Bio-Pharm Technology Co., Ltd. The raw sequencing data were processed for quality filtering and merging via Fastp and FLASH software. The optimized sequences were subsequently grouped into operational taxonomic units (OTUs) with a 97% similarity threshold via UPARSE 7.1 (Edgar, 2013).

### Statistical analysis

One-way analysis of variance (ANOVA) followed by Duncan’s test was performed via IBM SPSS 25 to assess treatment differences at a significance level of *p* < 0.05. Bar graphs were generated using Prism 9.5 software, and microbial data were analyzed using R software. Principal component analysis (PCA) of the bacterial and fungal communities and soil properties was conducted via the R package “vegan” (Wang et al., 2023). Two-factor networks and co-occurrence networks were constructed with the R package “psych” and visualized via Gephi software (Fan et al., 2018). Spearman correlation analysis between biochars and the microbial abundance of bacteria and fungi was conducted using the R package “ggcorrplot” (Liu et al., 2022). Graphs were combined using Adobe Illustrator 2021 software.

## Results

### Residual effects of biochar and nanomodified biochar on soil physicochemical properties

**FIGURE 1:**
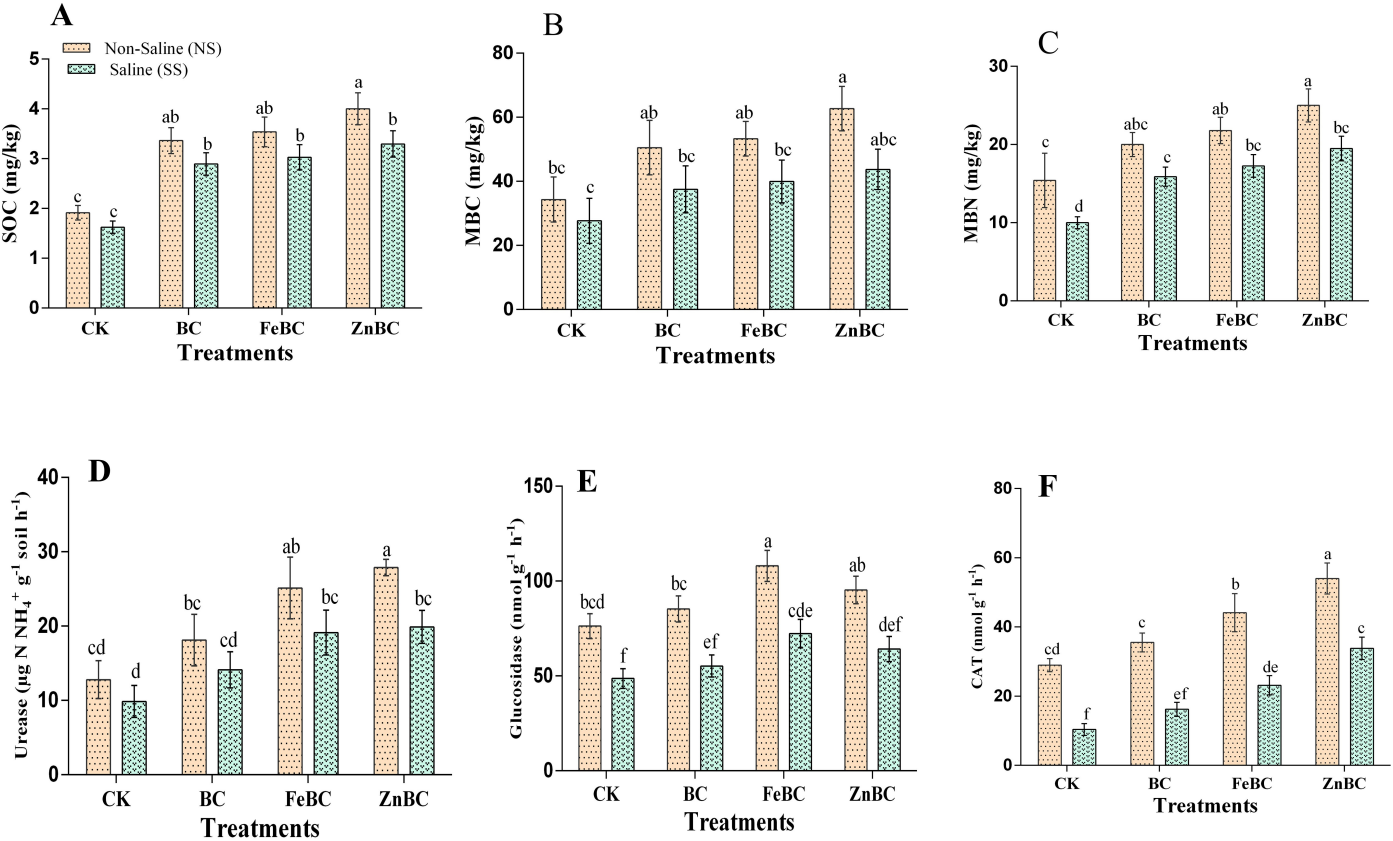
Effect of biochar (BC) and modified biochar (FeBC and ZnBC) on SOC, MBC, MBN, and enzymatic activities in non-saline (NS) and saline (SS) soil. Means values ± SD (*n* = 3); different letters represent statistically significant differences (least significant difference (DUNCAN) test, *p* ≤ 0.05). SOC, Soil Organic Carbon **(A)**; MBC, Microbial Biomass Carbon **(B)**; MBN, Microbial Biomass Nitrogen **(C)**; Urease **(D)**, Glucosidase: β-glycosidase **(E)** and CAT, Catalase **(F)**.

As shown in Table 1, salinity adversely affects soil nutrient availability and activities. However, the application of pristine biochar (BC) and nanomodified biochar (FeBC and ZnBC) significantly increased soil nutrient levels in both soils. The results revealed that BC significantly increased the pH in both SS and NS soils, whereas nanobiochar slightly decreased the pH. The soil organic matter (SOM) content increased by 14–20% in the biochar and nanomodified treatments in the NS and SS soils. SOC was also increased with the addition of biochar and modified biochar to the NS and SS soils (Figure 1A). The TN increased by 6.3–12.86% and 16–27% in the saline and NS soils, respectively, after biochar addition. The total phosphorus in the soil also increased by 8–20.3% in response to biochar and modified biochar application in the SS and NS soils. The modified biochar increased the total potassium (TK) content by 5% under both soil conditions. Similarly, the available potassium and phosphorus contents increased by 5–16% and 6–23%, respectively, after the addition of BC, FeBC, and ZnBC. Compared with that in the CK treatment, the NO_3_^–^-N concentrations in both nanomodified biochar treatments increased by 34% in the NS soil, whereas that in the saline soil increased by 17% after ZnBC treatment. Compared with that in the control, the concentration of ammonium nitrogen after the biochar and nanobiochar treatments increased by 7–13% in the NS soil and 7–17% in the SS soil. Soil MBC and MBN were significantly greater in the nanomodified biochar treatment group in the pristine biochar treatment group and the CK group under SS and NS soils (Figures 1B,C). Urease and catalase activities were higher in the ZnBC and FeBC modified biochars among other treatments under the NS and SS soil conditions (Figures 1D,E). Compared with the other biochars and CK, the Fe-modified biochar increased the activity of β-glucosidase in the NS and SS soils (Figure 1F). Both types of modified biochar promote soil physicochemical properties and activities in the NS and SS soils.

**TABLE 1:** Effect of biochar and Nano-modified biochar on the soil properties.

Treatments	Salt	Soil pH	SOM%	TN (g kg^–1^)	TP (g kg^–1^)	TK (g kg^–1^)	AP (mgkg^–1^)	AK (mgkg^–1^)	NO_3_ (mg kg^–1^)	NH_4_ (mg kg^–1^)
CK	NS	6.6 ± 0.23c	1.2 ± 0.11cd	0.26 ± 0.06a	0.33 ± 0.02b	0.76 ± 0.05abc	9.22 ± 0.77b	70.4 ± 5.3b	1.06 ± 0.09bc	3.06 ± 0.25a
BC		6.9 ± 0.20b	1.4 ± 0.24abc	0.28 ± 0.01a	0.36 ± 0.03ab	0.80 ± 0.06ab	9.74 ± 1.5ab	73.3 ± 7.7ab	1.38 ± 0.15a	3.31 ± 0.19a
FeBC		6.4 ± 0.11c	1.5 ± 0.18ab	0.29 ± 0.03a	0.39 ± 0.05a	0.81 ± 0.05a	10.1 ± 1.2ab	77.4 ± 6.5ab	1.41 ± 0.13a	3.39 ± 0.33a
ZnBC		6.5 ± 0.15c	1.5 ± 0.19a	0.29 ± 0.02a	0.39 ± 0.02a	0.81 ± 0.02ab	10.7 ± 1.1a	79.7 ± 11a	1.48 ± 0.19a	3.43 ± 0.38a
CK	SS	7.4 ± 0.17a	1.0 ± 0.07d	0.14 ± 0.01c	0.21 ± 0.01d	0.67 ± 0.08c	5.22 ± 0.5c	29.2 ± 6.3e	0.98 ± 0.07c	2.09 ± 0.22c
BC		7.5 ± 0.013a	1.2 ± 0.13bcd	0.16 ± 0.03bc	0.23 ± 0.03cd	0.69 ± 0.03c	5.61 ± 1.2c	36.1 ± 2.3e	1.11 ± 0.09b	2.25 ± 0.28bc
FeBC		7.3 ± 0.26a	1.3 ± 0.09abc	0.18 ± 0.06bc	0.26 ± 0.02cd	0.70 ± 0.04bc	5.89 ± 1c	40.4 ± 3.8cd	1.18 ± 0.13bc	2.44 ± 0.16bc
ZnBC		7.3 ± 0.22a	1.3 ± 0.21abc	0.18 ± 0.05b	0.25 ± 0.06c	0.71 ± 0.03abc	6.18 ± 0.89c	43.7 ± 3.6c	1.13 ± 0.07b	2.53 ± 0.41b

Different letters within the columns show a significant difference between treatments at the Duncun test (*P* < 0.05). NS represents no salt, and SS represents Salt Stress. OM, Soil Organic Matter; TN, Total Nitrogen; TP, Total Phosphorus; TK, Total Potassium; AP, Available Phosphorus; AK, Available Potassium; NO_3_^–^N, Nitrate Nitrogen; NH_4_^+^-N, Ammonium Nitrogen; CK, Control; BC, Biochar; FeBC, Iron Nano-Modified Biochar; ZnBC, Zinc Nano-modified Biochar.

### Effect of modified biochar on bacterial and fungal phyla

**FIGURE 2:**
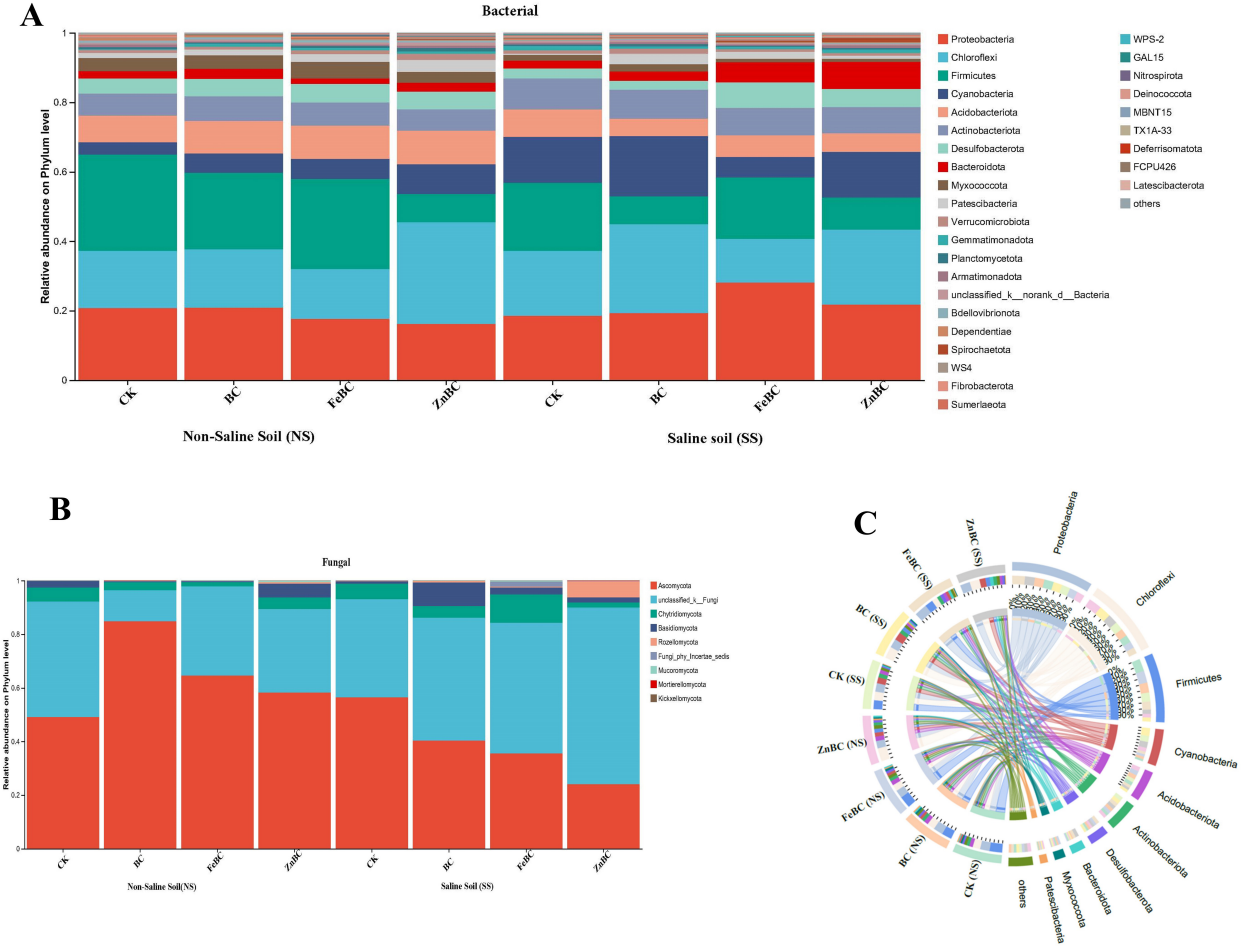
The changes in the abundance of the dominant phyla of bacterial **(A)**, fungal communities **(B)** and major bacterial communities contributing **(C)** in NS and SS soils after the addition of biochar and nano-modified biochar. SS, Saline Soil; NS, Non-saline Soil; CK, Control; BC, Biochar; FeBC, Iron Nano-Modified Biochar; ZnBC, Zinc Nano-modified Biochar.

The changes in the abundance of bacterial communities under different nanomodified biochar and pristine biochar applications in the SS and NS soils are shown in Figure 2A. Compared with that in the CK in the NS soil, the abundance of the phylum Proteobacteria in the ZnBC and FeBC treatments decreased by 15 and 17%, respectively. However, in the SS soil, compared with that in the CK, the abundance of Proteobacteria in the FeBC and ZnBC treatments increased by 25 and 22%, respectively. In the NS soil, the highest abundance of the phylum Chloroflexi was observed in the ZnBC treatment, whereas a lower abundance was detected in the FeBC treatment (Figure 2C). Compared with the CK treatment, the BC and ZnBC treatments presented 25 and 28% greater abundances of Chloroflexi, in the SS soil. The abundance of Firmicutes in the SS soil decreased in the BC and ZnBC treatments but significantly increased in the FeBC treatment. Compared with that in the CK treatment, the abundance of Cyanobacteria in the BC and ZnBC treatments increased in the NS soil, and ZnBC increased their abundance in the SS soil. The abundance of Bacteroides significantly increased with BC, FeBC, and ZnBC in the SS soil, but their abundance did not significantly differ in the NS soil.

There were significant changes in the abundance of the phyla Ascomycota, Chitridiomycota, Basidiomycota, and Rozellomycota across the different biochar treatments compared with those in the control in the SS and NS soils (Figure 2B). Ascomycota was the dominant fungal phylum in the SS and NS soils across all the treatments, constituting approximately 75–85% of the relative abundance. The relative abundance of Ascomycota increased with biochar application (BC > FeBC > ZnBC) in NS soil, whereas its relative abundance decreased with biochar application in saline soil compared with that in the control, and the lowest abundance was observed in the ZnBC treatment. The abundance of Basidiomycota increased with BC, FeBC, and ZnBC application in saline soil compared with that in NS soil. In the SS soil, the abundance of Chytridiomycota significantly increased in the FeBC treatments. Rozellomycota dominated only in the ZnBC treatment in the saline soil. The remaining phyla, including Chytridiomycota and Rozellomycota, contributed less than 5% of the overall fungal community.

### Changes in the microbial communities at the genus and class levels

**FIGURE 3:**
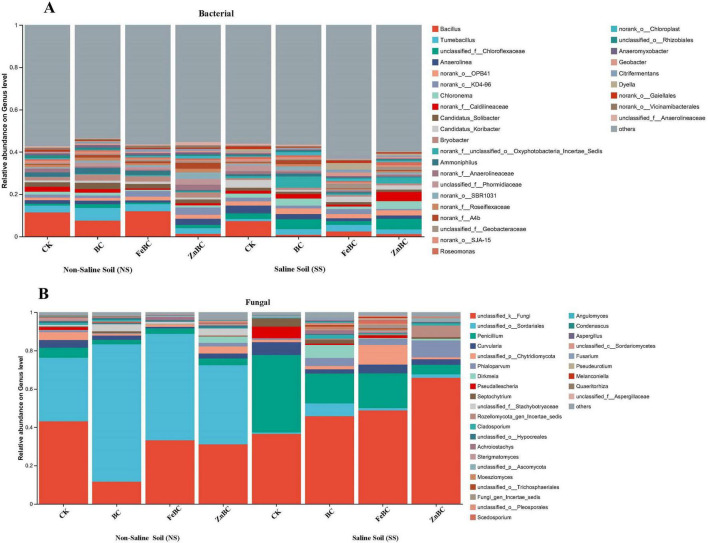
Effect of Biochar and Nano-modified biochar (FeBC, ZnBC) on the genus of the leading abundance of bacterial **(A)** and fungal communities **(B)** in saline and non-saline soils. SS, Saline Soil; NS, Non-saline Soil; CK, Control; BC, Biochar; FeBC, Iron Nano-Modified Biochar; ZnBC, Zinc Nano-modified Biochar.

**FIGURE 4:**
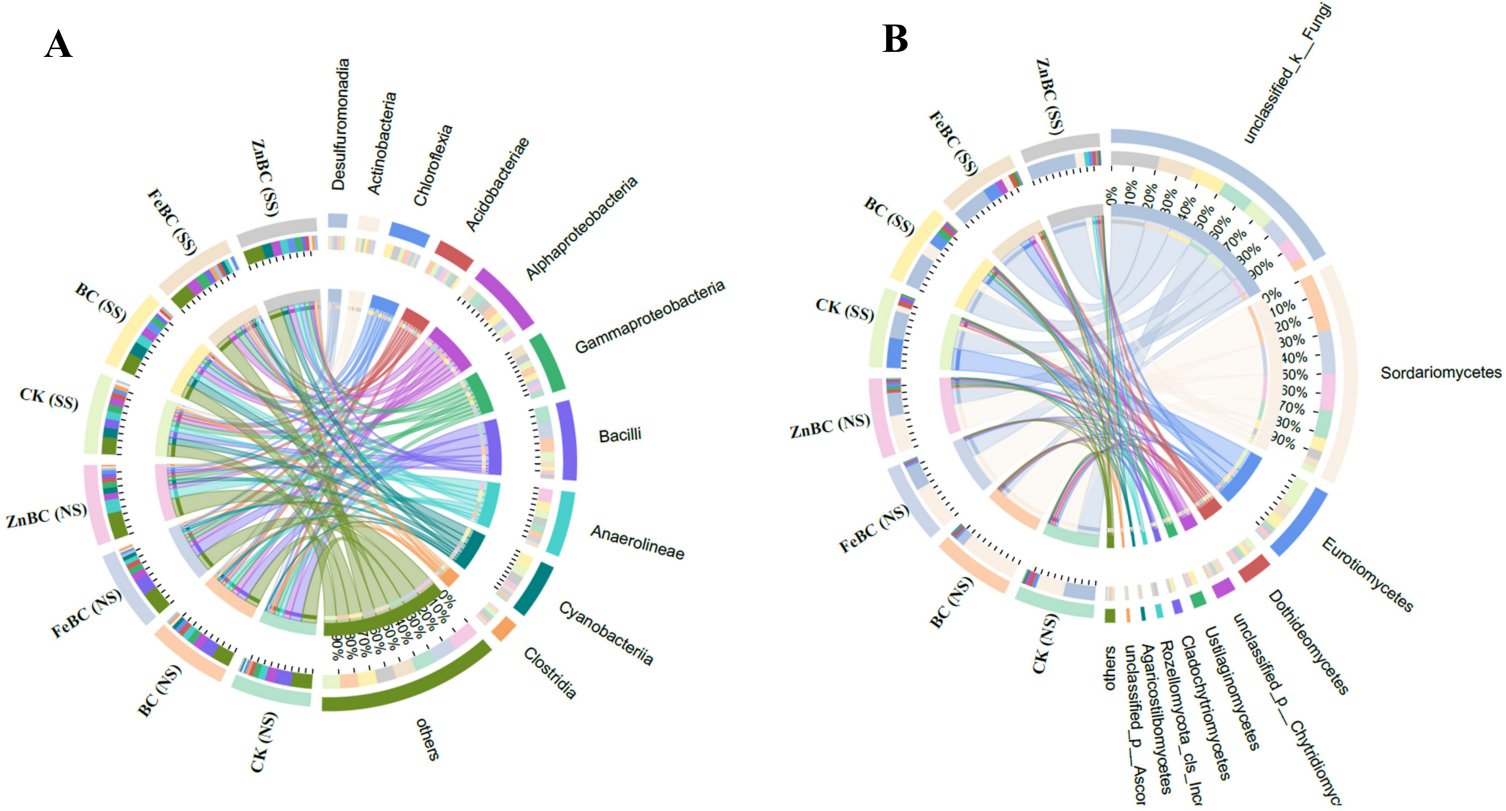
Circos plots show the distribution of microbial species at the class level of **(A)** Bacterial and **(B)** Fungal present in different treatments. One side of the circle plot shows treatments and their corresponding groups, while the other side shows the main dominant species, explaining the abundance distribution of different species within the treatments through the connection of inner ribbons. In the Circos treatments and species relationship diagram, the inner ring (Left Half Circle) represents the sample groups (different treatments in saline and non-saline soils), while the outer ring (Right Half Circle) represents the microbial species at the class level. SS, Saline Soil; NS, Non-saline Soil; CK, Control; BC, Biochar; FeBC, Iron Nano-Modified Biochar; ZnBC, Zinc Nano-modified Biochar.

The BC and Fe- and Zn-modified nanobiochars significantly enhanced the bacterial and fungal communities at the genus level in both the NS and the SS soils (Figure 3A). The abundance of the genus *Bacillus* slightly increased under the FeBC treatment in NS soil, whereas compared with those in the control, while the abundances in the BC and ZnBC treatments significantly decreased. The abundance of *Tumabacillus* significantly increased in all the biochar treatments, regardless of the soil conditions. Additionally, the abundance of *Anaerolinea* significantly increased in all the biochar treatments in the NS soil. The application of BC, FeBC, and ZnBC slightly affected the abundance of *norank-o-opb41* in the NS soil, whereas it significantly increased only after ZnBC treatment in the saline soil compared with that in the CK. The abundance of *Chloronema* increased in the BC and ZnBC treatments in the SS soil. *Alphaproteobacteria* and *Gammaproteobacteria* were the predominant classes in the saline soil in FeBC, whereas *Bacilli* was the leading predominant class in the NS soil (Figure 4A).

The relative abundances of fungal genera subjected to different biochar treatments under both the SS and NS conditions are depicted in a bar plot in Figure 3B. *Penicillium* abundance was greater in the saline soil than in the NS soil, whereas it was lower in the BC, FeBC, and ZnBC treatments. The relative abundances of other genera, such as *Curvularia*, *unclassified_p_Chytridiomycota*, and *Phialoparvum*, trended to vary across the treatments, but the overall abundances of these genera were greater in the saline soil than in the NS soil. The most dominant class in the NS soil was Sordariomycetes, whereas the class Eurotiomycetes was the major class in the SS soil (Figure 4B).

### Network analysis of microbial communities at the phylum and genus levels

**FIGURE 5:**
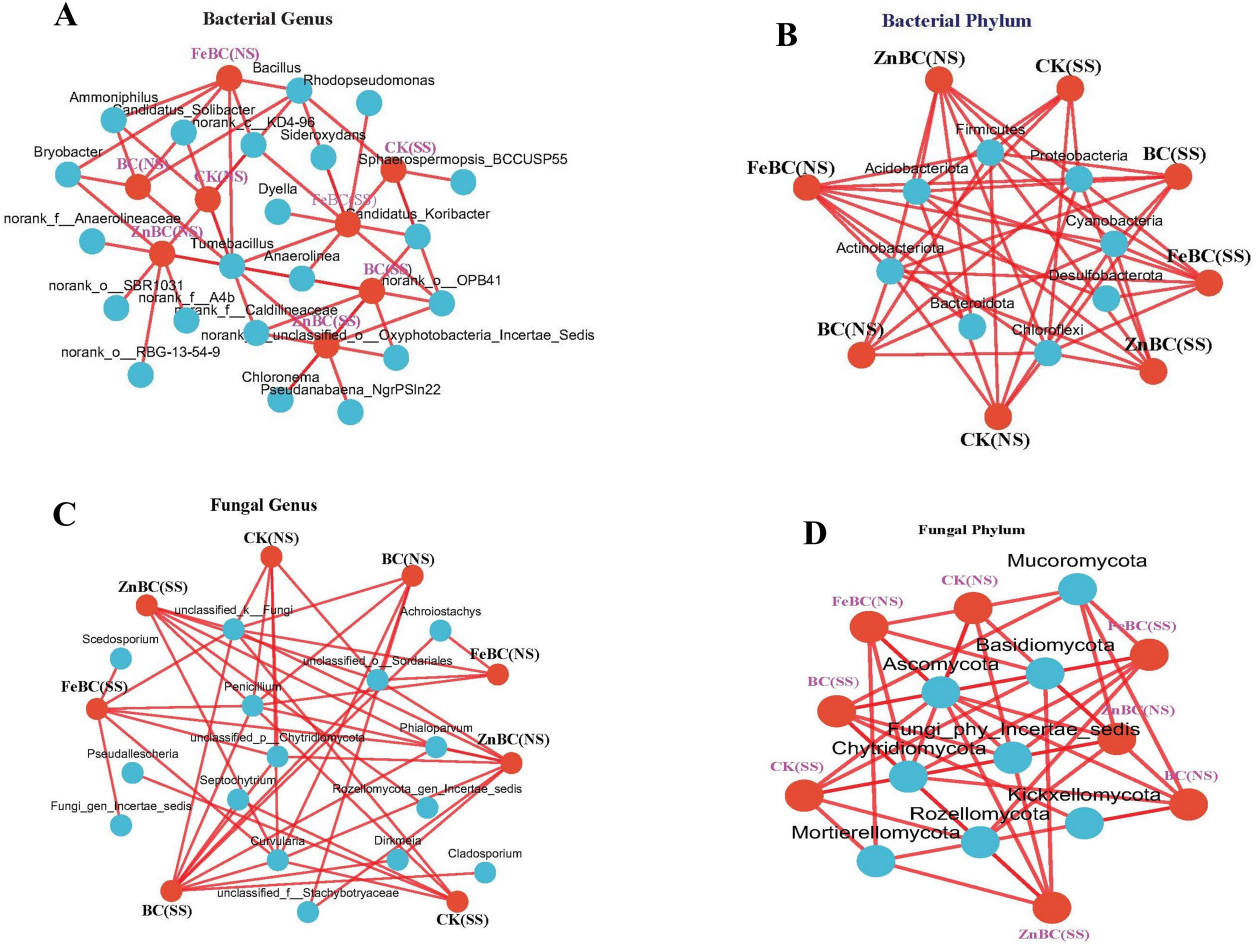
The co-occurrence pattern between the microbial community of bacteria **(A,B)** and fungi **(C,D)** at the phylum and genus level in biochar and nano-modified biochar treatments (FeBC, ZnBC) under saline and non-saline soil conditions. Blue nodes indicate individual species, while red nodes indicate treatments, whereas edges connecting the nodes show the relationship between them. The strength of the relationship is often represented by the thickness of the edge. Nodes with thicker edges between them have a stronger relationship. NS represents non-saline soil and SS means saline soil. SS, Saline Soil; NS, Non-saline Soil; CK, Control; BC, Biochar; FeBC, Iron Nano-Modified Biochar; ZnBC, Zinc Nano-modified Biochar.

Network analysis revealed complex interactions among the top ten bacterial phyla and treatments (Figures 5A,C). Proteobacteria, Chloroflexi, Firmicutes, Cyanobacteria, Acidobacteria, and Actinobacteria presented strong associations with the BC, FeBC, and ZnBC treatments in the non-saline soil, suggesting their central roles and improvements in the microbial community. In the saline soil, BC was strongly associated with changes in the abundances of Proteobacteria, Chloroflexi, Firmicutes, and Actinobacteria. Ascomycota, Basidiomycota, Mucoromycota, Chytridiomycota, Rozellomycota, and Fungi_Phy_Incertae_sedis were strongly associated with the BC, FeBC, and ZnBC treatments. Kickxellomycota was related only to the BC treatments in the NS and SS soils, and Mortierellomycota presented the fewest connections with BC and ZnBC in both soil types. The network also clearly differed between the SS and NS soil treatments, highlighting the significant impact of salinity on the bacterial and fungal community structure.

Network analysis is used to visualize the relationships between different microbial species (or genera) within a microbial community (Figures 5B,D). The network analysis results revealed that the genus *Bacillus* was strongly related to BC and FeBC in both soils. Changes in *Ammoniphilus* and *Bryobacter* were strongly related to the NS soil, whereas no connection was detected in the saline soil. *Rhodopseudomonas* was connected to the FeBC treatment in the saline soil, whereas the other treatments had weak connections. *Norank_o__OPB41* was strongly related to all the treatments in the saline soil. *Norank_o__SBR1031*, *norank_o__RBG-13–54–9*, *norank_f__Anaerolineaceae*, and *Anaerolinea* were strongly related to the ZnBC treatment in the non-saline soil. Network analysis revealed a complex interplay between fungal genera and treatments (Figure 3). *Penicillium* was strongly associated with NS soil. *Septochytrium* was connected to only the BC treatment in the SS soil, whereas *Scedosporium* was connected to the FeBC treatment in the SS soil. *Phialoparvum* had stronger connections with ZnBC in both soils and with the BC and FeBC treatments in the SS soil.

### Heatmap relationships of microbial phyla

**FIGURE 6:**
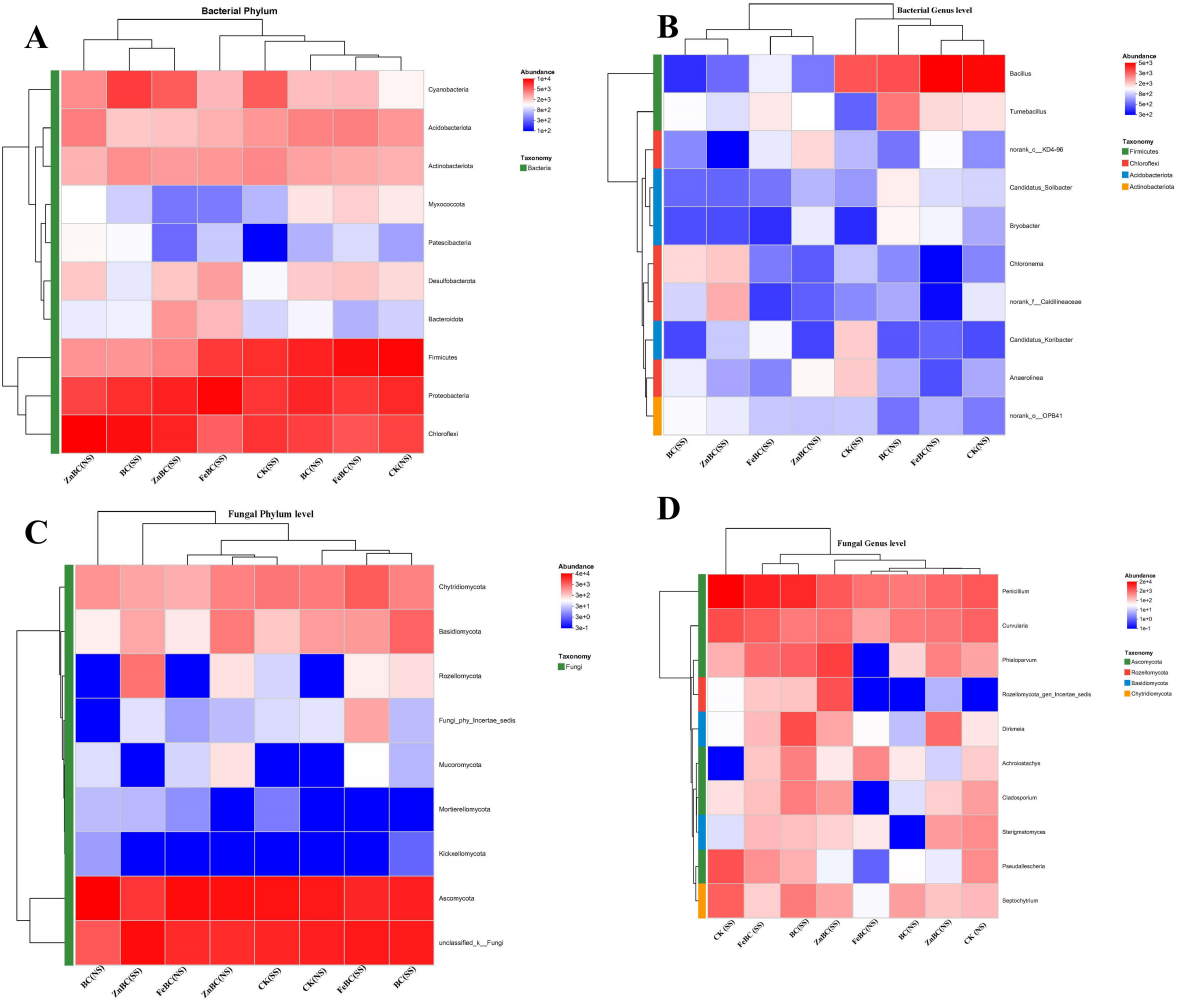
Heatmap showing the distribution of dominant bacterial and fungal communities under biochar and nano-modified biochar treatments in saline and non-saline soils. **(A)** Bacterial phylum level, **(B)** bacterial genus level, **(C)** fungal phylum level, and **(D)** fungal genus level. The abscissa represents different treatments, and the ordinate represents microbial taxa. Color gradients indicate relative abundance, as shown in the legend. NS represents non-saline soil, and SS represents saline soil. CK, Control; BC, Biochar; FeBC, Iron nano-modified biochar; ZnBC, Zinc nano-modified biochar.

A clustered heatmap was constructed and revealed the relationships of the top 10 bacterial phyla with the different biochar treatments under both soil conditions (Figure 6A). The bacterial phyla presented relatively high abundances and clustered together, suggesting strong similarities in their abundance patterns across the different treatments and soil types due to the dominance of Proteobacteria and the high relative abundances of Chloroflexi and Firmicutes in most samples. Compared with those of the other phyla, the Cyanobacteria phylum, with high abundance clustered together, indicating a response to the BC and ZnBC treatments under saline soil conditions. Compared with those in the SS soil, the Acidobacteria phylum in the NS soil presented high cluster abundances in all the treatments. The remaining phyla (Desulfobacteria, Bacteriodota, and Myxococcota) with the lowest abundances formed a distinct cluster. The results of the clustered heatmaps revealed the relationships of the different phyla with the biochar and modified biochar treatments in both soil types (Figure 6C). Ascomycota were more abundant and clustered together, suggesting a strong similarity in their abundance patterns across the BC, FeBC, and ZnBC treatments in the SS and NS soils. The Chytridiomycota and Basidiomycota phyla with high abundances were clustered together, indicating similar responses to FeBC and ZnBC in NS soil compared with those in SS soil. The remaining phyla (Rozellomycota, Fungi_phy_Incertae_sedis, Mucoromycota, Mortierellomycota, and Kickxellomycota) with the lowest abundances formed a distinct cluster (Figure 6C). These findings suggest that biochar treatments have some influence on the bacterial community, but their effects are more pronounced than those of salinity. The interaction effect between salinity and biochar likely influences the overall community structure.

### Relationships of microbial genera with modified biochar addition

Based on the results of the heatmap analysis, *Bacillus* was highly abundant in non-saline soil, and compared with BC, Zn BC, and FeBC resulted in a greater cluster. *Tumebacillus* was also significantly abundant in the NS soil in the BC treatment. The results further revealed that the abundances of *Bryobacter*, *Candidatus_Solibacte*, and *Chloronema* were relatively consistently lows across most treatments, indicating that they were less prominent. These data suggest notable microbial diversity and differences in community composition across the samples. Heatmap analysis revealed that salinity was the primary driver of fungal community composition at the genus level, with clear separation between the SS and NS soils (Figure 6B). *Penicillium* and *Curvularia* appeared to be the dominant genera across the BC, FeBC, and ZnBC treatments in both the saline and NS soils, indicating their ecological significance in these environments. The row dendrogram suggests potential ecological similarity or functional redundancy between *Penicillium* and *Curvularia*, as they cluster together. Other genera, such as *Phialoparvum*, *Rozellomycota*, *Cladosporium*, and *Sterigmatomyces*, exhibited more distinct abundance patterns and were more abundant in the saline soil than in the NS soil. *Pseudallescheria* and *Septochytrium* also clustered together and were less abundant in the NS group (Figure 6D). The column dendrogram highlights the strong influence of salinity on the fungal community, with clear separation between the SS and NS samples.

### Relationships between soil properties and major bacterial and fungal abundances

**FIGURE 7:**
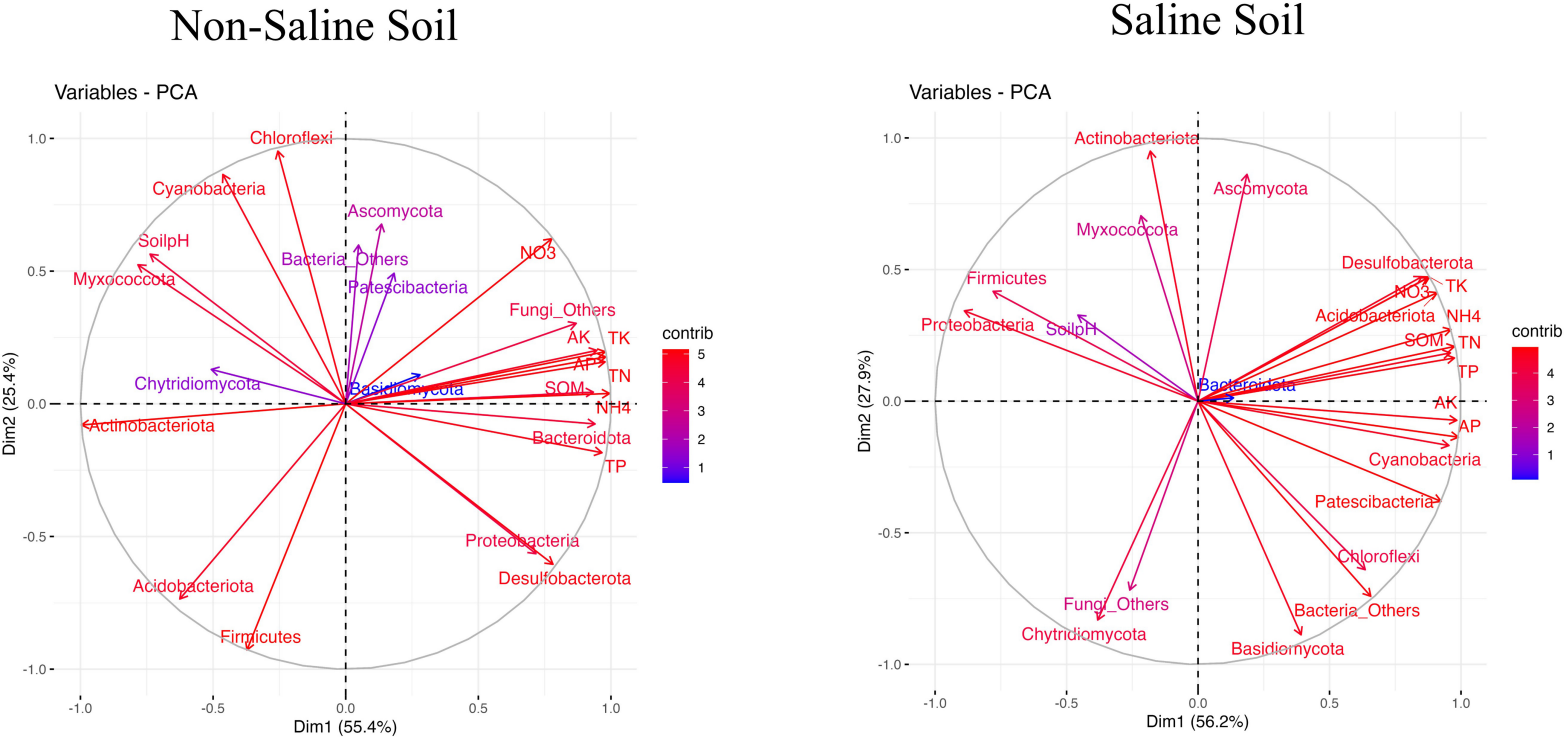
Principal Component Analysis (PCA) biplot, between soil physicochemical properties and microbial community composition. Soil Organic Matter (SOM); Total Nitrogen (TN); Total Phosphorus (TP); Total Potassium (TK); Available Phosphorus (AP); Available Potassium (AK); Nitrate (NO_3_); Ammonia (NH_4_).

The relationships between soil physicochemical properties and microbial community composition were explained in NS and SS soils via principal component analysis (PCA). The NS biplot shows 80.8% of the total variance, Dim1 (55.4%) and Dim2 (25.4%), whereas the SS soil biplot displays 91.1% of the total change, Dim1 (56.2%) and Dim2 (27.9%), providing a robust representation of the data (Figure 7). The strong separation along Dim1 suggests that this dimension captures the primary gradient of variation in the dataset. The variables with longer vectors along the axis, such as Proteobacteria, Desulfobacterota, and Bacteroidota, and soil properties, such as TN, SOM, TP, and NH_4_, were significant contributors to this variation. Similarly, Dim2 further explained the significant contributions of Chloroflexi, Cyanobacteria, and soil pH to the variation along this axis. The results also revealed positive correlations between TN, AP, TK, AK, Proteobacteria, Desulfobacterota, and Bacteroidota.

## Discussion

### Changes in soil properties after the addition of nanomodified biochar

Salinity negatively affects soil properties, leading to reduced nutrient availability and impaired plant growth due to osmotic stress and ion toxicity. The introduction of biochar, especially when modified with nanoparticles such as Fe and Zn, enhances soil health by improving key physicochemical properties. The addition of BC significantly increased the pH of both soils due to the alkaline nature of the biochar, which increased the soil pH (Gao et al., 2019). The increase in pH is attributed to the basic cations on the biochar surface, which is consistent with the findings of Khan et al. (2022a,b), who reported a pH increase following the application of rice straw biochar. Furthermore, a slight decrease in the pH of the modified biochar treatments (FeBC) was detected at either level, which was due to the initial low pH of the modified biochar. Soil organic matter (SOM), a vital measure of soil fertility, is greatly enhanced by the application of BC (Khan et al., 2022a,b). Here, our results revealed a significant increase in the soil organic matter and AP contents with the addition of Fe- and Zn-modified biochar (Table 1). Previous reports have indicated that the improvement in SOM caused by biochar provides essential substances for microbial growth (Ren et al., 2018). Similarly, the application of ZnBC enriched with ZnO nanoparticles resulted in the most pronounced effects among all the treatments. These results indicate that zinc not only improves nutrient uptake but also significantly increases nitrogen availability, as evidenced by increases in both nitrate (NO_3_^–^-N) and ammonium nitrogen (NH_4_^+^-N) levels (Xia et al., 2023; Abbas et al., 2024). The possible mechanism for the reduction in pH is associated with the effects of these treatments (FeBC and ZnBC), which create a more favorable environment for nutrient solubility and availability.

The mechanisms through which the treatments improve soil fertility are multifaceted. Both FeBC and ZnBC improve the cation exchange capacity of the soil, allowing for better retention of essential nutrients such as nitrogen, phosphorus, and potassium (Ghassemi-Golezani and Rahimzadeh, 2022) or the release of organic acids during biochar decomposition (Hafeez et al., 2022). The reduced pH could also be partly due to Zn, as it can generate zinc hydroxide complexes that influence the acidity (Xia et al., 2024). A decreased pH can increase the availability of certain nutrients, particularly phosphorus, in alkaline soils. The increase in SOM with ZnBC and FeBC application can be attributed to the high carbon content of biochar, which is resistant to decomposition (Ghassemi-Golezani and Rahimzadeh, 2022). The potential of zinc to stimulate microbial activity leads to increased organic matter turnover and accumulation (Lv et al., 2022). Similarly, the substantial 37.59% increase in TN with ZnBC can be explained by the high surface area and porosity of the biochar, which can adsorb and retain nitrogen compounds (Issa, 2022; Lv et al., 2022). Soil enzymes are mainly produced by microorganisms, and their activity is closely linked to microbial health. In the present research, catalase activity significantly increased with the addition of ZnBC and FeBC biochars. Wojewodzki et al. (2022) as well as Nie et al. (2018) also observed a significant increase in enzymatic activity after the addition of biochar. Furthermore, previous research has shown that the activity of soil enzymes would be affected by the surface area of biochar, mainly due to its retention potential and subsequent capacity for changing or rotating the active site of enzymes (Elzobair et al., 2016; Foster et al., 2018). The soil urease directly participates in the transformation of nitrogen-containing organic matter to N available to plants in the soil, and its active level is an indicator of the soil nitrogen level (Yan and Quan, 2013), involving in the redox reaction beneficial to plant metabolism (Oladele, 2019). In addition, the overall activities of soil enzymes at different soil layers and between various biochar treatments vary with the amount added, which is also common in previous studies.

### Effects of biochar and modified biochar on bacterial abundance

Nano-BC has garnered increasing attention for its potential to remediate contaminated soil, as it possesses unique characteristics, such as a large surface area and effective hydrodynamic dispersion, which distinguish it from regular BC. The composition of the microbial community may significantly vary between the biochar-amended and control treatments due to high salinity (Kaoping et al., 2019; Fan et al., 2020). In high-salinity soil, the growth of microbes is suppressed, and the diversity of microbial communities is reduced (Feng et al., 2023). Only those microbes that can withstand high salt concentrations can live in high-salinity soil (Liu et al., 2023). The predominant saline–alkalinesoil microorganisms are Gemmatimonadetes, Chloroflexi, Acidobacteria, Firmicutes, Proteobacteria, and Actinobacteria. Currently, the abundances of phyla Chloroflexi, Actinobacteria, Bacteroidota, and Proteobacteria are strongly related to salinity and are abundant in saline soil (Figure 2). Multiple bacterial taxa have been discovered and reported in response to biochar, control, and nanobiochar treatments. In the present investigation, the dominant phyla were Chloroflexi (primarily *norank_c__KD4-96* and *Chloronemanorank_o__OPB41*), Bacteroidota, Proteobacteria, and Firmicutes (*Tumebacillus* and *Bacillus* genera) (Figure 2). According to current studies, the Bacteroidota and Proteobacteria phyla are widely distributed and highly adaptable to saline environments (Wang et al., 2022). Because Actinobacteria and Chloroflexi are highly resilient to saline conditions, they are abundant and have been reported in various saline alkaline environments (Gobalakrishnan, 2022). Furthermore, phylotypes are linked to Bacteroidetes and Firmicutes, which are relatively highly salt-tolerant (Ren et al., 2018).

Biochar and nanomodified biochar increased the proportions of salt-resistant microbes of the phyla Chloroflexi, Bacteroidota, Actinobacteria, and Proteobacteria (Figure 2), consequently mitigating saline-alkali biochar stress and enhancing the metabolism and relationships of these microbes with plants. This investigation revealed that the abundances of Acidobacteria, Bacteroidetes, and Firmicutes increased in high-salt environments (Yang et al., 2021). Similarly, as mentioned above, to increase salinity stress, bacterial communities as well as certain phyla, Cyanobacteria, *Bacillus*, and Bacteroidetes, exhibit strong structural responses and tolerance mechanisms (Zhang M. et al., 2021). These microbes, which can withstand highly saline conditions, are highly beneficial for plant growth and development. These microbes can minimize the adverse effects of salinity, which increases agricultural production in saline soils (Abd El Daim et al., 2015; Egamberdieva et al., 2019). With the addition of biochar, the abundances of Bacteroidota and Proteobacteria are clear indicators that microbial activity increases due to the presence of organic carbon supplied by the addition of biochar to the soil (Ling et al., 2022). The addition of FeBC lower soil pH and consequently enhanced the abundance of Acidobacteriota, which proliferate best in acidic condition (Wu et al., 2019). Based on these evidences, we identified a greater number of core nodes bacterial coexisting networks under biochar treatments compared with CK. Moreover, the probiotic Bacteroidota, a type of *Sphingomonas* used in FBC treatment as a biomarker, is responsible for inhibiting antioxidation activity and soil pathogens (Deng et al., 2022; Wang et al., 2022). Our results suggest that reducing soil salt and increasing organic carbon and available nutrients under modified biochar application could provide a better habitat environment and nutrients for bacteria, which benefits soil health and plant growth.

### Impact of modified biochar on the fungal community

Ther application of biochar significantly influenced the fungal community composition in both soil types, particularly at the phylum level. Ascomycota was the dominant fungal phylum, which is consistent with its known adaptability to various soil conditions (Treseder and Lennon, 2015). In the present study, the BC, FeBC, and ZnBC treatments increased the abundance of Ascomycota in the NS soil, likely due to improvements in soil structure, water retention, and nutrient availability, which promoted fungal proliferation (Gul et al., 2015). Conversely, in the saline soil, compared with the control treatment, the application of BC, FeBC, and ZnBC reduced the abundance of Ascomycota, suggesting that salinity stress, possibly exacerbated by biochar-induced changes in soil chemistry, negatively affects growth (Zhang G. et al., 2021; Baldrian et al., 2022). The relative abundance of Unclassified_k_Fungi increased with biochar application in both soil types, with higher levels in the saline soil, indicating the presence of stress-tolerant fungi that benefit from the role of biochar in mitigating extreme conditions (Zhang G. et al., 2021; Han et al., 2024). Additionally, Chytridiomycota and Basidiomycota were more prevalent in the saline soils than in the NS soils under biochar and modified biochar treatments, suggesting that biochar may increase the growth of opportunistic and ligninolytic fungi that contribute to organic matter decomposition and nutrient cycling (Runnel et al., 2025). These shifts in fungal community composition may be attributed to the influence of biochar on soil physicochemical properties, microbial competition, and salinity alleviation, which affect fungal functional dynamics (Lehmann et al., 2011; Baldrian et al., 2022). Overall, biochar amendments differentially modulated fungal communities under both SS and NS conditions, with potential implications for soil health and resilience (Khan et al., 2024).

The application of biochar significantly altered the fungal community composition at the genus level, highlighting its role in shaping microbial ecology in both saline and non-saline soils. *Unclassified_k_Fungi* was the dominant fungal group across all the treatments, with a notably greater abundance in saline soil under biochar application, particularly in BC, FeBC, and ZnBC, suggesting that biochar provides a suitable habitat for stress-tolerant fungal taxa (Treseder and Lennon, 2015; Feng et al., 2023; Li et al., 2023). The second most dominant genus,*_Sordariales*, was more abundant in NS soils, especially in the BC treatment, indicating its preference for more favorable soil conditions and its role in organic matter decomposition and nutrient cycling (Gul et al., 2015). *Penicillium* abundance increased significantly in the saline soil with biochar and modified biochar application, suggesting that *Penicillium* has a potential ecological function in saline environments because of its known salt tolerance, bioactive metabolite production, and plant growth-promoting properties (Li et al., 2021; Yin et al., 2021). Other fungal genera, including *Curvularia*, and *Phialoparvum*, exhibited varying trends across treatments, but their overall abundances were greater in saline soils, suggesting that biochar may increase fungal diversity by mitigating salt stress and creating microhabitats favorable for stress-adapted fungi (Baldrian et al., 2022; Li et al., 2023). The increased abundances of *Penicillium* in saline soil under biochar treatment may be attributed to improved soil properties, reduced salinity stress, and enhanced microbial interactions, as biochar is known to increase the soil cation exchange capacity, organic matter content, and microbial habitat stability (Lehmann et al., 2011). These findings suggest that biochar and nanomodified biochar amendments play crucial roles in modulating fungal community composition by promoting decomposers and nutrient-cycling fungi in NS soil while enhancing salt-tolerant and stress-adapted fungi in saline environments, ultimately improving soil microbial resilience and ecosystem functioning (Wang et al., 2020).

## Conclusion

This research revealed that biochar and nanomodified biochar significantly alleviated salinity stress in paddy soil and improved soil health and fertility. In our study, biochar and nanomodified biochar affected the soil pH and increased the soil organic matter, SOC, and available and total nutrients present in the soil. Nanomodified biochar improved the abundances of bacteria at the phylum and genus levels in the SS and NS soils. In addition, the results of this study suggest that these genera may play a role in regulating the abundances of other members of the bacterial community. The network structure provides insights into potential ecological associations and functional relationships among different genera and phyla. Considering its multiple roles in improving saline soil, supplying nutrients to plants, and regulating beneficial bacteria, FeBC could play a crucial role in enhancing food security and promoting sustainable agricultural development in arid and semiarid regions. Additionally, this research introduces new perspectives for the use of modified biochar and emphasizes the potential for developing biochar microelement composite materials to improve agricultural yields and promote sustainability. Future research will be conducted to investigate the mechanistic pathways through functional gene and metagenomic analysis which modified biochars influence plant, soil, and microbe interactions under salinity stress.

## Data Availability

The raw data supporting the conclusions of this article will be made available by the authors, without undue reservation.
